# IL-10 provides cardioprotection in diabetic myocardial infarction via upregulation of Heme clearance pathways

**DOI:** 10.1172/jci.insight.133050

**Published:** 2020-09-03

**Authors:** Rajesh Gupta, Lijun Liu, Xiaolu Zhang, Xiaoming Fan, Prasanna Krishnamurthy, Suresh Verma, Jörn Tongers, Sol Misener, Nikita Ashcherkin, Hongliu Sun, Jiang Tian, Raj Kishore

**Affiliations:** 1 Division of Cardiovascular Medicine, Department of Medicine, University of Toledo College of Medicine, Toledo, Ohio, USA.; 2 Feinberg Cardiovascular Research Institute, Northwestern University Feinberg School of Medicine, Chicago, Illinois, USA.; 3 Department of Biomedical Engineering and; 4 Division of Cardiovascular Disease, Department of Medicine, University of Alabama at Birmingham, Birmingham, Alabama, USA.; 5 Mid-German Heart Center, Department of Internal Medicine III, Division of Cardiology, Angiology and Intensive Medical Care, University Hospital Halle, Martin-Luther-University, Halle (Saale), Germany.; 6 Department of Pathology, University of Toledo College of Medicine, Toledo, Ohio, USA.; 7 Center for Translational Medicine, Temple University School of Medicine, Philadelphia, Pennsylvania, USA.

**Keywords:** Cardiology, Therapeutics, Cardiovascular disease, Cytokines, Diabetes

## Abstract

Diabetes is a risk factor for myocardial infarction, and outcomes after myocardial infarction are worse among diabetics compared with nondiabetics. Diabetes is associated with impaired Heme clearance. Here, we determined whether heme toxicity and impaired heme clearance contribute to diabetic myocardial infarction injury and assessed IL-10 as a therapeutic agent for diabetic myocardial infarction. Plasma-free hemoglobin was significantly elevated in diabetic mice compared with nondiabetic mice after myocardial infarction. Infarct size had strong correlation to the level of plasma-free hemoglobin. Hemoglobin and reactive iron deposition within the infarct zone were also demonstrated in diabetic MI. IL-10 significantly reduced infarct size and improved cardiac function in diabetic mice. Moreover, IL-10 improved capillary density, reduced apoptosis, and decreased inflammation in the border zone of the infarcted hearts, findings that were partially inhibited by Tin protoporphyrin (a heme oxygenase-1 inhibitor). IL-10 upregulated CD163, the hemoglobin:haptoglobin scavenger receptor, and heme oxygenase-1 in THP-1–derived and primary human CD14^+^ macrophages. IL-10 significantly protected against ischemic injury when HL-1 cardiomyocytes were cotreated with hemoglobin. Together, our findings indicate that IL-10 is cardioprotective in diabetic myocardial infarction via upregulation of heme clearance pathways. These findings implicate heme clearance as a potentially novel therapeutic direction for diabetic myocardial infarction.

## Abstract 2

Interleukin-10 promotes heme clearance pathways and is cardioprotective in a murine diabetic myocardial infarction model.

## Graphical abstract

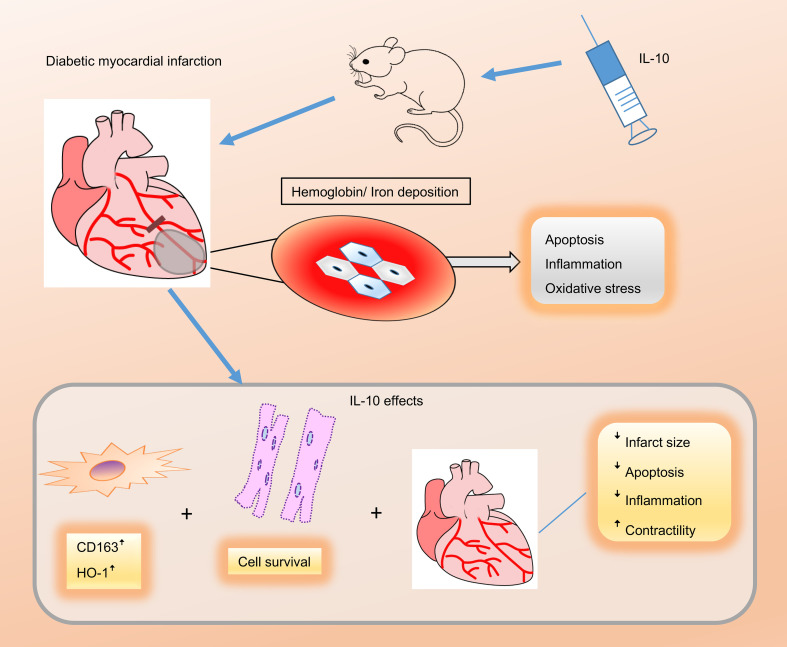

## Introduction

Myocardial infarction (MI) is a common problem resulting in clinical sequelae, including heart failure and death (1). Diabetes is a powerful risk factor for atherosclerosis and MI (2). Furthermore, diabetic patients are known to have more severe injuries after MI, as well as more complications (3, 4). However, the mechanisms underlying this observation are not well understood; therefore, specific therapeutic strategies for diabetic MI (DM MI) have not been elucidated.

Recent data have implicated myocardial hemorrhage in the pathogenesis of MI. Newer methods using cardiac MRI have allowed in vivo imaging of myocardial hemorrhage, which was previously unappreciated (5–7). Furthermore, cardiac MRI studies have demonstrated areas of microvascular destruction and hemorrhage within the infarct core (8). Heme has been recognized as a damage associated molecular pattern (DAMP) activing numerous adverse biological responses (9). Prior studies have demonstrated that IL-10 robustly increases the expression of the heme:haptoglobin scavenger receptor, CD163, on macrophages (10, 11). In addition, IL-10 is known to upregulate heme oxygenase-1 (HO-1), the major intracellular enzyme for heme degradation (12).

Prior studies have demonstrated that diabetes impairs heme clearance mechanisms (13–15). Diabetes is known to downregulate CD163, the hemoglobin:haptoglobin (Hb:haptoglobin) scavenger receptor. IL-10 is a potent antiinflammatory cytokine and suppresses various proinflammatory mediators (16). Recent studies have demonstrated a protective role for IL-10 in cardiac dysfunction and remodeling following acute MI through inhibition of cardiac inflammation and fibrosis (17). However, the therapeutic effect of systemic IL-10 on DM MI and the underlying molecular mechanisms are unknown. We assessed the role of myocardial hemorrhage and free heme in the pathogenesis of DM MI and investigated the role of IL-10 in cardioprotection after DM MI.

## Results

### Increased plasma-free Hb and myocardial infarct size in diabetic mice.

**Figure 1:**
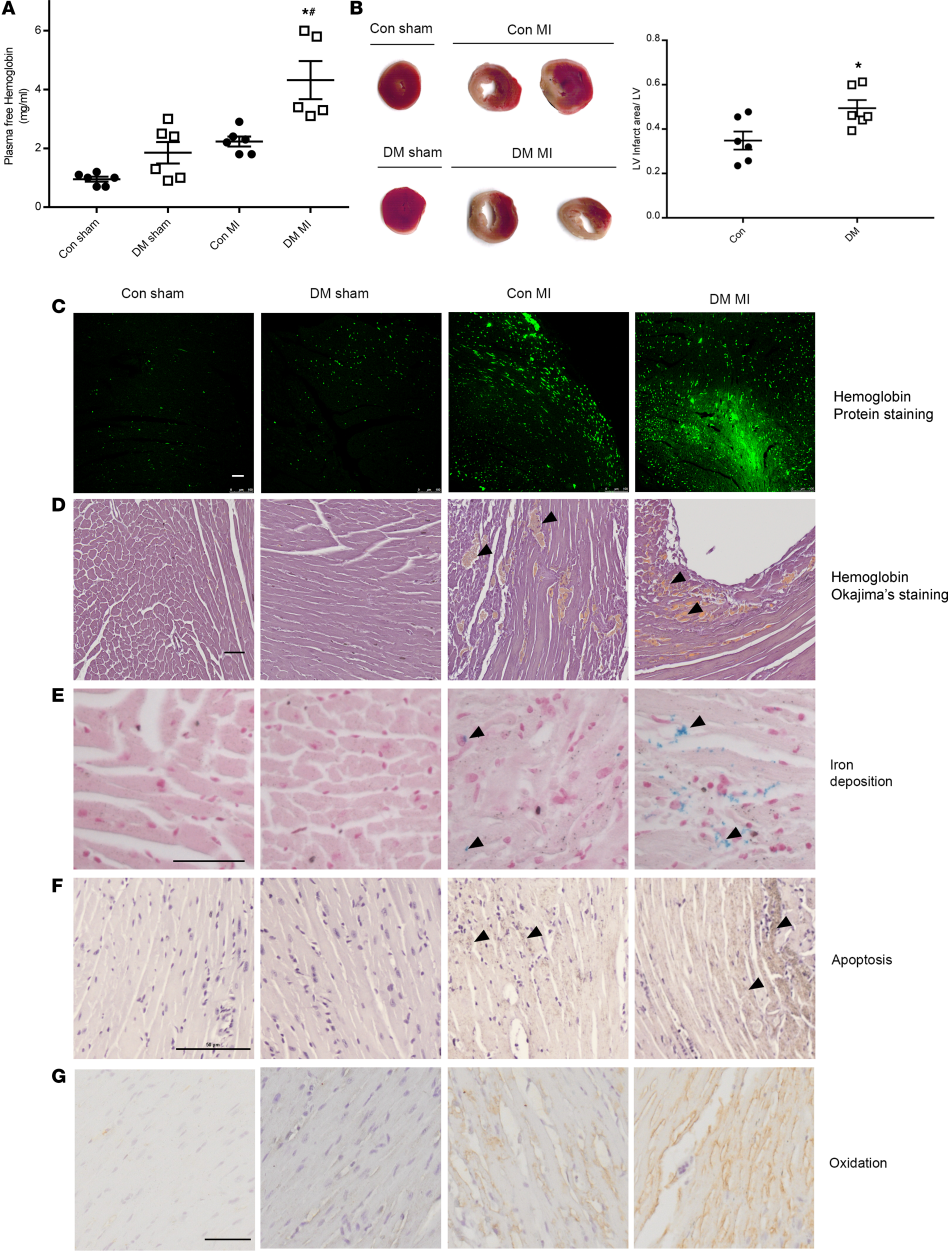
Characterization of toxicity of free hemoglobin in diabetic MI. Control and STZ-induced diabetic mice were subjected to MI by ligation of left anterior descending coronary artery. (**A**) Plasma-free hemoglobin was increased in DM mice at 24 hours after MI. *n* = 5–6 per group, 1-way ANOVA, followed by Tukey’s test. *P < 0.05 vs. DM sham; ^#^*P* < 0.01 vs. Con MI. (**B**) TTC staining of heart sections at 24 hours after MI shows larger infarct size in DM mice. Left: representative images; Right: percentage of LV infarction area to LV total area. *n* = 6 per group, *t* test, *P < 0.05 vs. Con. (**C** and **D**) Hemoglobin protein fluorescence staining and Okajima’s staining show hemoglobin deposits in the heart 72 hours after MI. (**E**) Accumulation of iron in the infarct zones in the ferric (Fe^3+^) oxidation state as determined by Prussian blue staining. (**F**) Localization of apoptosis in Con MI and DM MI determined by immunohistochemical analysis of paraffin-embedded heart section using cleaved caspase-3 Ab. (**G**) The extent of 4-hydroxynonenal immune reactivity as shown in brown in the infarct zones. Scale bars: 50 μm. Arrowheads indicate the positive reactions.

Diabetic condition was induced by low-dose streptozotocin (STZ) (50 mg/kg, i.p.) daily for 5 days. Mice underwent left anterior descending (LAD) ligation surgery 2 weeks after the last STZ dose, when fasting blood glucose levels reached a mean of approximately 22.2 mmol/L (400 mg/dL) (Supplemental Figure 1A; supplemental material available online with this article; https://doi.org/10.1172/jci.insight.133050DS1). Twenty-four hours after MI was induced, plasma-free Hb levels were measured. MI increased levels of plasma-free Hb (Figure 1A, *P* < 0.05, MI vs. sham). DM MI mice had significantly higher plasma-free Hb compared with control (Con) MI mice (Figure 1A, *P* < 0.05, DM MI vs. Con MI). 2,3,5-Triphenyl-tetrazolium chloride (TTC) staining measures heart tissue viability and was used to evaluate infarct size. TTC staining at 24 hours after MI demonstrated larger infarct size in DM MI compared with Con MI (Figure 1B, *P* < 0.05, Con vs. DM). Pearson correlation coefficient for plasma-free Hb and infarct size was 0.82 (*P* < 0.05), indicating that there was a strong positive correlation between plasma-free Hb and infarct size.

Hb immunofluorescence and Okajima staining were performed to document Hb deposition in the infarction zones (Figure 1, C and D). Perls’ Prussian blue staining of heart tissues revealed positive ferric iron deposition in the infarct zone, further confirming the presence of free hemoglobin (Hb) in the infarct zone (Figure 1E). Next, we measured heme-scavenging proteins, hemopexin and haptoglobin. Plasma hemopexin and haptoglobin responded to MI and dramatically increased at 24 hours after MI (Supplemental Figure 1, B and C). Hepatic *hp* mRNA was significantly increased after MI both in control and diabetic mice (Supplemental Figure 1D). However, there were no differences of hemopexin and haptoglobin between DM MI mice and nondiabetic MI mice.

To explore the mechanism of free Hb toxicity, apoptosis was measured by active caspase-3 IHC staining of heart tissues. There were more positive active caspase-3 cells in the infarct zone and border zone in diabetic mice compared with control mice (Figure 1F). Furthermore, lipid peroxidation assessed by 4-hydroxynonenal (4-HNE) was present largely in the infarct zone in diabetic mice compared with control mice (Figure 1G).

Immunofluorescence staining of CD163, CD68, and nuclei (DAPI) of heart tissues (Supplemental Figure 2), indicates that CD68^+^ macrophages were increased in the infarct border zone after MI. The number of CD68^+^ cells per high-power field (hpf) in DM MI hearts was higher than the number in Con MI group (Supplemental Figure 2B, left panel). The number of CD163^+^ cells was significantly increased in the border zones after MI in both control and diabetic mice. However, the number of CD163^+^ cells per hpf in DM MI was significantly reduced compared with Con MI (Supplemental Figure 2B, middle panel). The ratio of CD163/CD68, an indicator of M2 polarization, was significantly reduced in DM MI hearts compared with Con MI hearts (Supplemental Figure 2B, right panel).

### IL-10 reduced MI size and improved cardiac function in diabetic mice.

**Figure 2:**
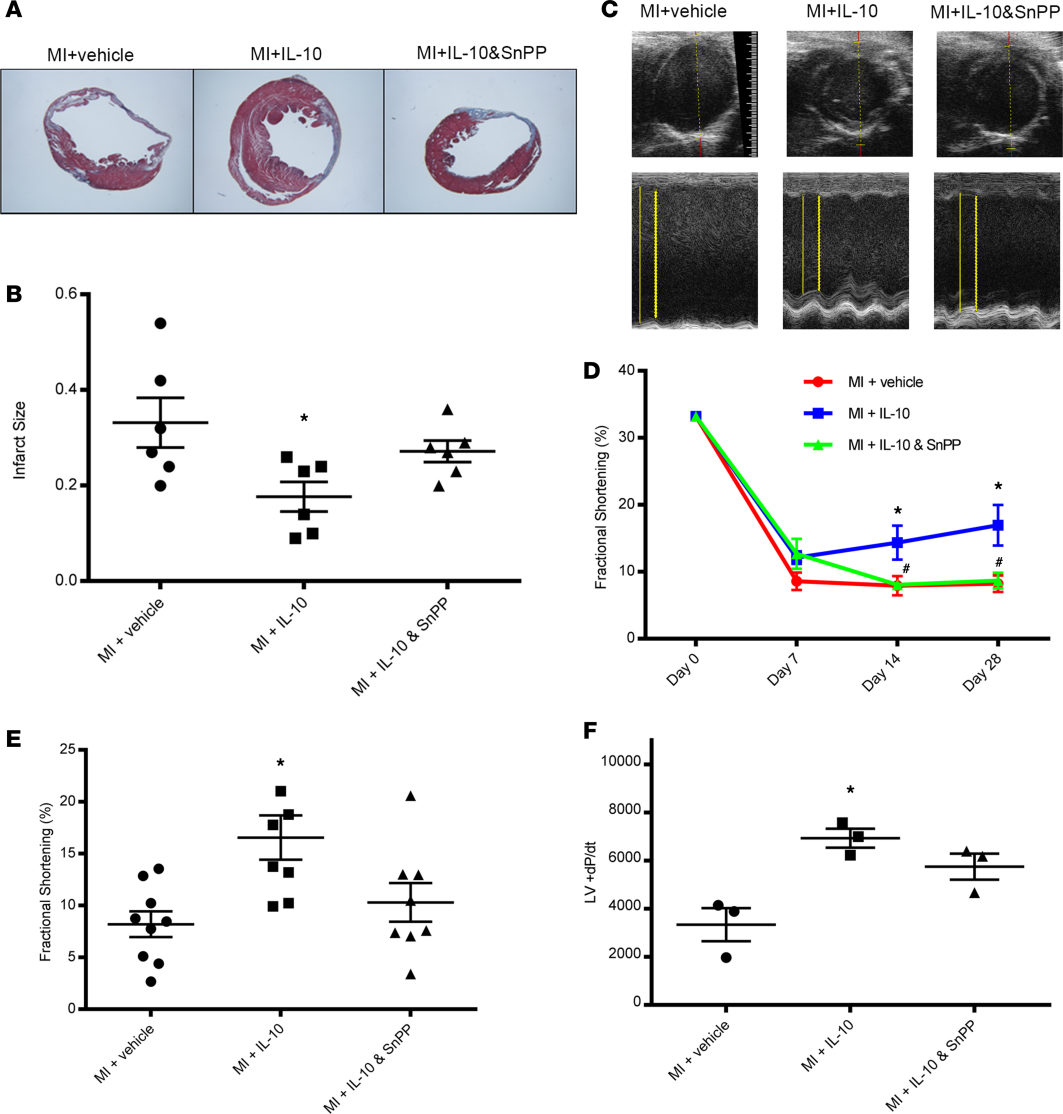
IL-10 reduced infarct size and improved cardiac function after MI in diabetic mice. (**A**) Representative images from Masson trichrome staining of diabetic mouse hearts on day 28 after MI. (**B**) Quantitative data of infarct size of left ventricles. One-way ANOVA, followed by Tukey’s test. *n* = 6. *P < 0.05 vs. MI + vehicle. (**C**) Representative images from 2D and M-mode of echocardiography. Solid lines indicate left ventricular diameter at end-diastole; dotted lines indicate left ventricular diameter at end-systole on M-mode images. **(D**) Fractional shortening of left ventricles by echocardiography at baseline (day 0) and day 7, 14, and 28 after MI; 2-way ANOVA followed by Tukey’s test. *n* = 6-9, *P < 0.05 vs. MI + vehicle, ^#^*P* < 0.05 vs. MI + IL-10. (**E**) Quantitative data of fractional shortening of left ventricles on day 28 after MI; 1-way ANOVA, followed by Tukey’s test; *P < 0.05 vs. MI + vehicle. (**F**) Left ventricular positive inotropy measured by an LV pressure-volume catheter directly inserted into the LV. One-way ANOVA followed by Tukey’s test. *n* = 4, *P < 0.05 vs. MI +vehicle.

Next, since DM MI demonstrated increased plasma-free Hb and larger infarct size, we focused our subsequent experiments on DM MI. We sought to determine if IL-10 could provide cardioprotection in DM MI. Diabetic mice were treated with vehicle (1% BSA), IL-10 (50 μg/kg, s.c.), or IL-10 (50 μg/kg, s.c.) plus Tin protoporphyrin (SnPP, a HO-1 inhibitor) (25 mg/kg, i.p.) at days 0, 1, 3, 5, and 7 after MI. Myocardial infarct size on heart tissue sections was measured by Masson’s trichrome staining on day 28 after MI. As depicted in Figure 2, A and B, IL-10 significantly reduced myocardial infarct size (*P* < 0.05, vs. vehicle); IL-10 plus SnPP partially reversed the protective effect of IL-10 on infarct size (*P* > 0.05 vs. vehicle). Serial echocardiography at baseline and days 7, 14, and 28 after MI (Figure 2, C–E) showed IL-10 improved left ventricular (LV) fractional shortening compared with the vehicle group at day 14 and day 28 (*P* < 0.05 vs. MI + vehicle), and this protective effect of IL-10 was inhibited by SnPP (*P* < 0.05 vs. IL-10). Furthermore, LV function measured by invasive hemodynamic assessment at day 28 also demonstrated improved LV + dP/dt with IL-10 treatment (Figure 2F).

### IL-10 increased HO-1 expression, improved capillary density, and reduced apoptosis in myocardium after MI in diabetic mice.

**Figure 3:**
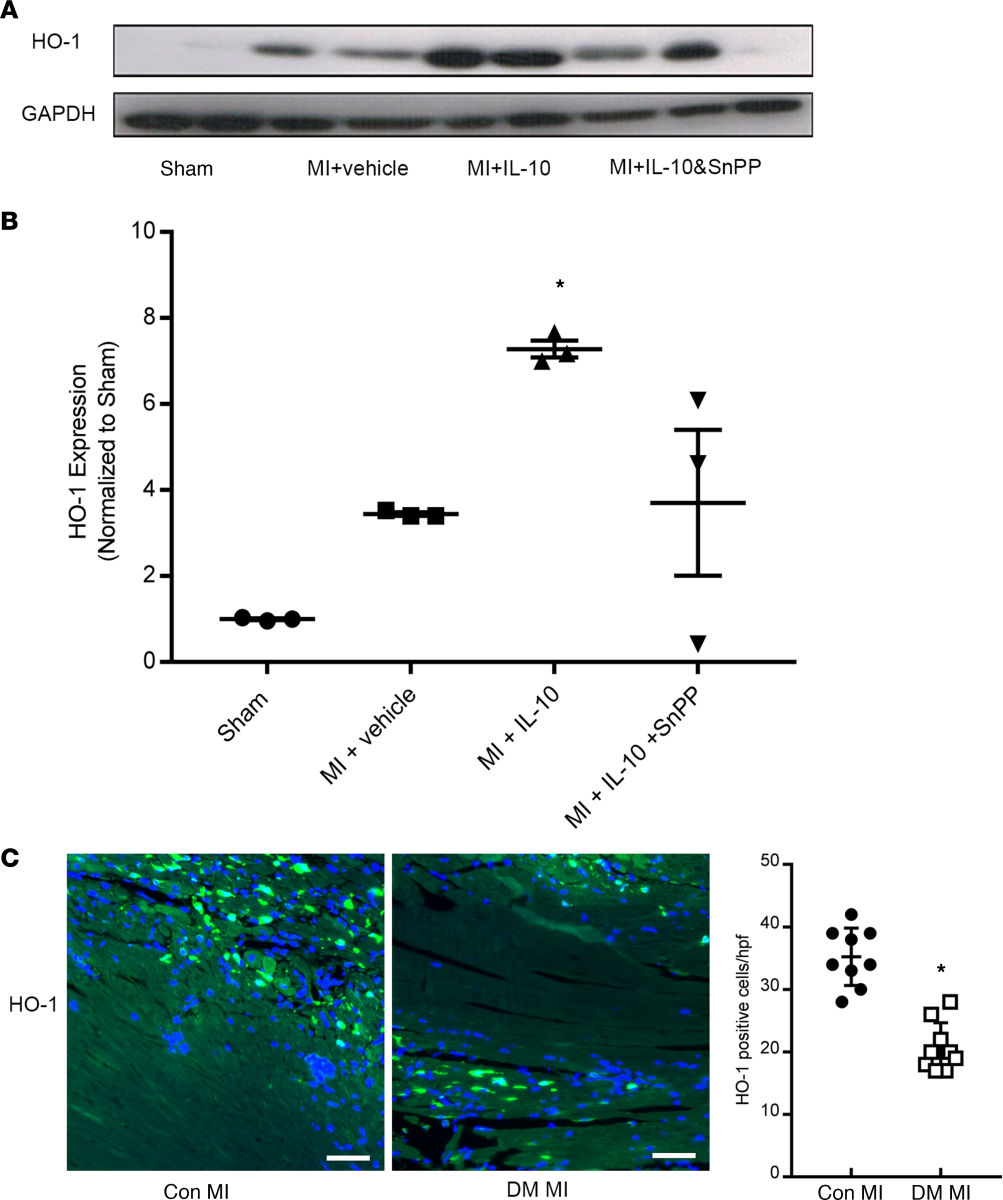
Systemic IL-10 induced HO-1 expression in the infarcted hearts in diabetic mice. Diabetic mouse left ventricular tissues on day 3 after MI, including the infarct and border zone, were lysed and measured for HO-1 protein expression by Western blot. GAPDH was used as a loading control. (**A**) Representative blots of HO-1 and GAPDH. (**B**) Quantitative analysis of HO-1 expression in diabetic hearts after MI. One-way ANOVA followed by Tukey’s test. *n* = 3 per group, *P < 0.05 vs. sham. (**C**) Left: immunofluorescence staining of HO-1 (green fluorescence) in the border zones of heart sections on day 3 after MI in control and diabetic mice. Blue color is the DAPI staining. Scale bar: 50 μm. Right: quantitative data of HO-1^+^ cells on day 3 after MI for control and diabetic mouse hearts. Independent *t* test. *n* = 9, *P < 0.05 vs. Con MI.

**Figure 4:**
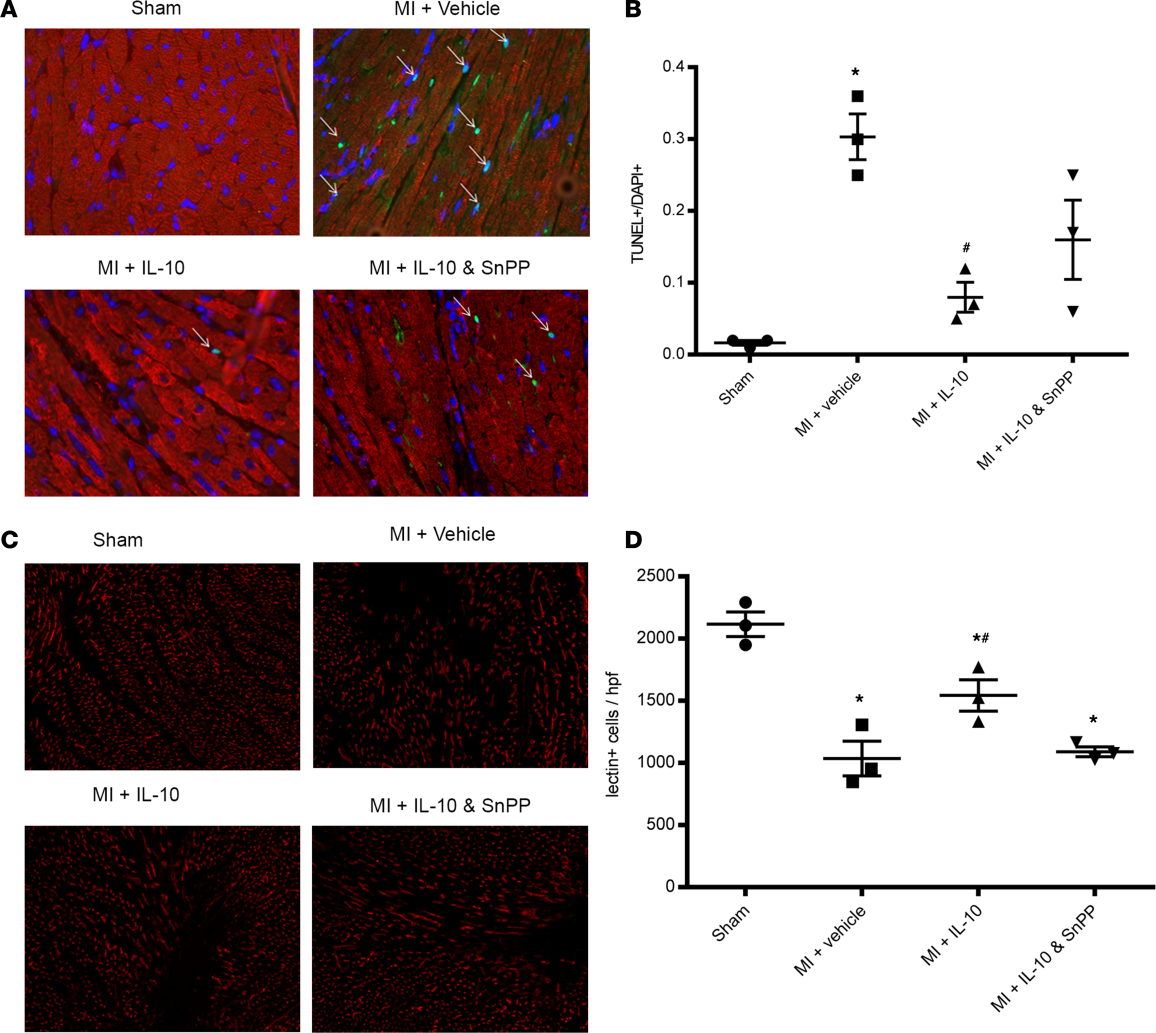
IL-10 reduced cardiomyocyte apoptosis and increased capillary density in the infarct border zone after MI in the diabetic mice. (**A**) Representative images of TUNEL assay (total original magnification, ×400). Arrows indicate TUNEL^+^ cells. Cardiomyocyte apoptosis was determined by the ratio of TUNEL^+^ cells to total cells. (**B**) Quantitative data of TUNEL assay. One-way ANOVA, followed by Tukey’s test; *n* = 3, *P < 0.05 vs. sham, ^#^*P* < 0.05 vs. MI + vehicle. (**C**) Representative images of lectin-positive cells in hearts (total original magnification, ×100). Mice were injected with BS-1 lectin via the tail vein at least 10 minutes before sacrifice, and tissue sections were stained for lectin expression. (**D**) Quantitative data on capillary densities of infract border zone on day 3 after MI. One-way ANOVA, followed by Tukey’s test; *n* = 3, *P < 0.05 vs. sham, ^#^*P* < 0.05 vs. MI + vehicle.

Next, we sought to measure myocardial HO-1 expression and other parameters of myocardial infarct biology, including apoptosis and capillary density (Figure 3 and Figure 4). As depicted in Figure 3, MI increased myocardial HO-1 expression compared with the sham group. Furthermore, IL-10 treatment significantly induced HO-1 expression, and this enhancement of IL-10 on HO-1 was partially blocked by HO-1 inhibitor SnPP. We also sought to compare expression of HO-1 in the infarcted heart in diabetic and Con MI. Fluorescence staining of HO-1 in heart sections revealed increased HO-1^+^ cells in the border zone both in Con MI and DM MI hearts, compared with sham (Figure 3C). However, the number of HO-1^+^ cells per hpf was significantly lower in DM MI hearts compared with Con MI hearts (Figure 3C). Taken together, these data demonstrate that DM MI has impaired HO-1 regulation, and IL-10 can robustly induce cardiac HO-1 expression in DM MI.

Cardiomyocyte apoptosis was analyzed by TUNEL assay and costaining with sarcomeric α-actinin in heart sections with focus on the infarct border zone. As depicted in Figure 4, A and B, DM MI significantly increased myocyte apoptosis (*P* < 0.05 vs. sham). IL-10 treatment significantly reduced apoptotic cells in the infarct border zone (*P* < 0.05 vs. MI + vehicle). The effect of IL-10 plus SnPP was less effective on apoptosis compared with IL-10 alone, although there is no statistical difference in apoptosis between IL-10 plus SnPP and IL-10, or between IL-10 plus SnPP and vehicle (*P* > 0.05).

As shown in Figure 4C, mice were injected with BS-1 lectin on day 3 after MI in order to assess the capillary density in the infarct border zone, a measure of adaptive angiogenesis. As depicted in Figure 4D, DM MI significantly reduced capillary density in the infarct border zone (*P* < 0.05 vs. sham). IL-10 treatment increased capillary density of the infarct border zone (*P* < 0.05 vs. MI + vehicle), and this effect was partially blocked by IL-10 plus SnPP (*P* > 0.05 vs. MI + vehicle).

### Effect of IL-10 on inflammation in diabetic infarct hearts.

**Figure 5:**
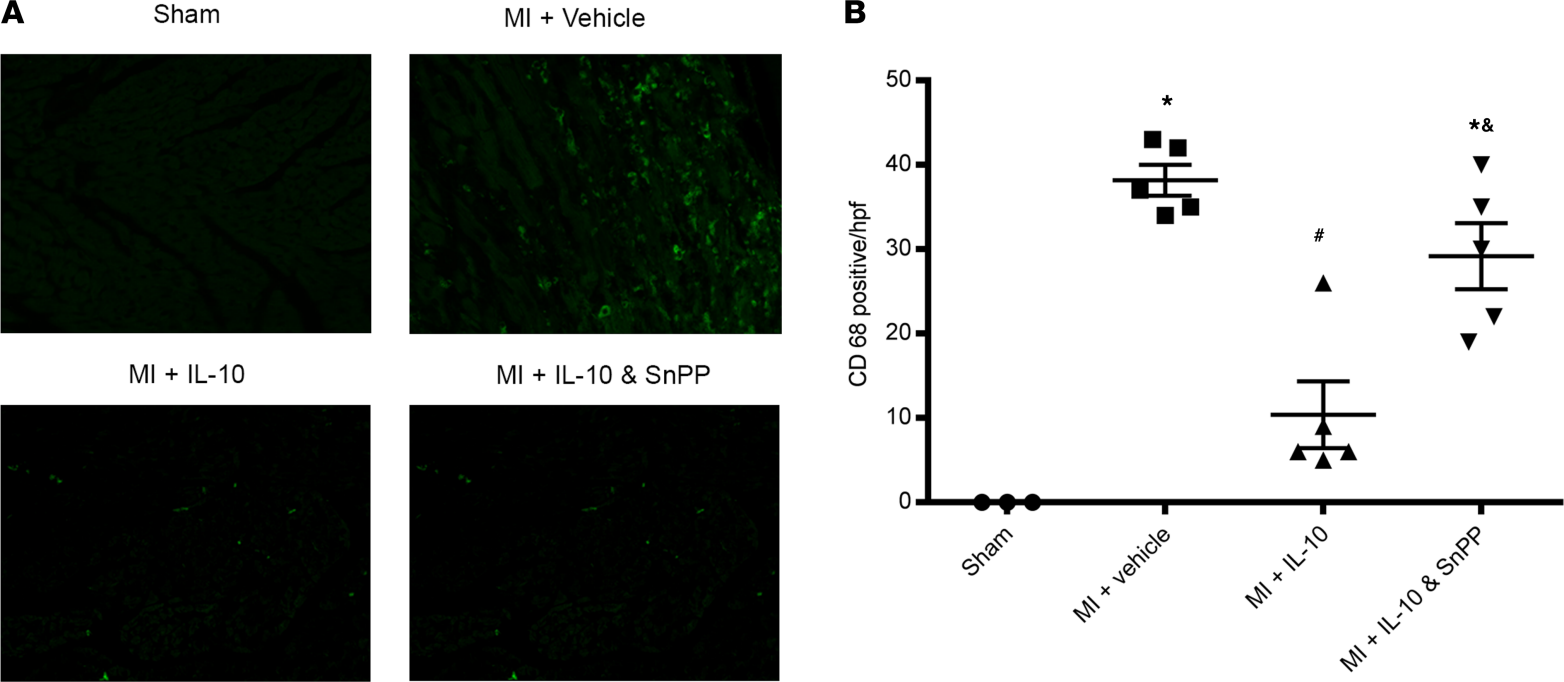
IL-10 suppressed inflammation after MI in diabetic mouse hearts. (**A**) Immunofluorescence staining of inflammatory cell (CD68^+^, green fluorescence) in the heart sections on day 3 after MI (total original magnification, ×400). (**B**) Quantitative data of infiltrating CD68^+^ cells on day 3 after MI. One-way ANOVA, followed by Tukey’s test; *n* = 3–5, *P < 0.05 vs. sham, ^#^*P* < 0.05 vs. MI + vehicle, ^&^*P* < 0.05 vs. MI + IL-10.

As depicted in Figure 5, A and B, increased infiltration of CD68^+^ cells (macrophage and monocyte) in the infarct border zone on day 3 after MI was observed in the MI + vehicle group (*P* < 0.05 vehicle vs. sham). IL-10 treatment significantly suppressed CD68^+^ cells in the infarct border zone (*P* < 0.05 vs. vehicle). IL-10 plus SnPP significantly blocked the effect of IL-10 on infiltration of CD68^+^ cells (*P* < 0.05, IL-10 plus SnPP vs. IL-10).

**Figure 6:**
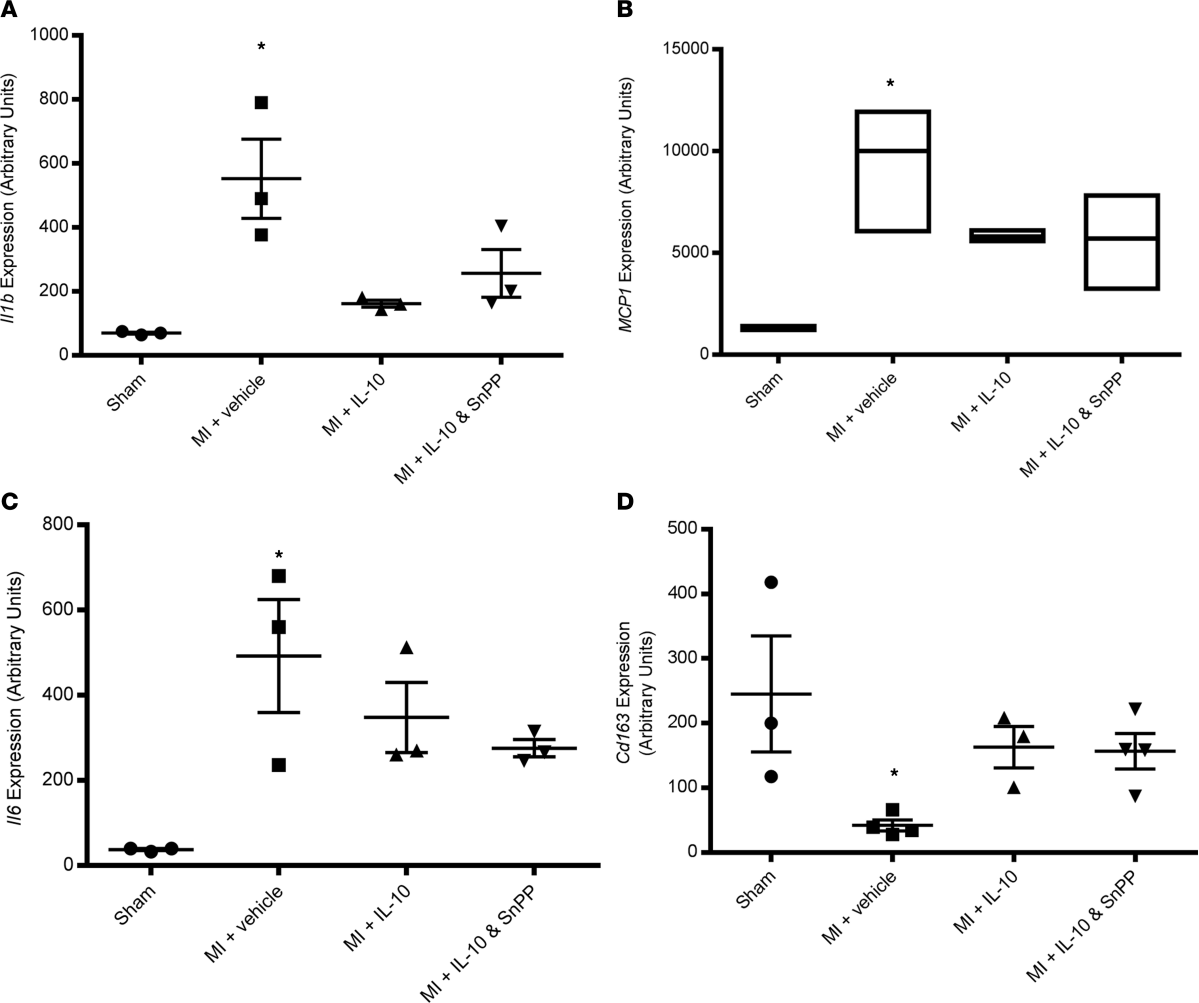
mRNA expression of proinflammatory cytokines and chemokine in the border zone of the LV infarct on day 3 after MI in diabetic mice. (**A–D**) Quantitative data of *Il1b* (**A**), *MCP*1 (**B**), *Il6* (**C**), and *cd163* (**D**). One-way ANOVA, followed by Tukey’s test, was applied to analyze the data of *Il1b*, *Il6*, and *cd163*, and scatter plots are presented. Nonparametric Kruskal-Wallis test was conducted in the data of *MCP*1, followed by Dunn’s test. A box plot is presented. *n* = 3 per group, *P < 0.05 vs. sham.

Next, we assessed inflammatory gene expression in the border zone of infarcted hearts. MI induced proinflammatory cytokines and chemokines (*Il1b*, *MCP1*, and *Il6*) in diabetic hearts on day 3 after MI (*P* < 0.05 vs. sham, Figure 6). IL-10 had a trend in reducing *Il1b* and *MCP1* expression compared with MI + vehicle. However, there was no statistical difference between IL-10 and IL-10 plus SnPP groups.

### Scavenger receptor CD163 expression in DM MI mice.

Free heme, which is mainly derived from heme-containing proteins such as Hb, is a potent oxidant and has proinflammatory properties. Plasma-free Hb was increased after DM MI (Figure1A). CD163 is the scavenger receptor for Hb:haptoglobin complexes and is expressed on macrophages. It has been reported that monocyte CD163 expression is reduced in diabetes (13, 14) and CD163 is strongly induced by IL-10 in macrophages (10, 11). Therefore, we sought to determine if IL-10 can induce CD163 expression in DM MI. As depicted in Figure 6D, MI in diabetic mice significantly reduced *cd163* mRNA expression in the heart on day 3 after MI (*P* < 0.05 vs. sham). IL-10–treated mice had less reduction in CD163 mRNA expression and were comparable with sham. IL-10 plus SnPP had a similar effect to IL-10 alone.

### Effect of IL-10 on macrophage CD163 and HO-1 expression.

**Figure 7:**
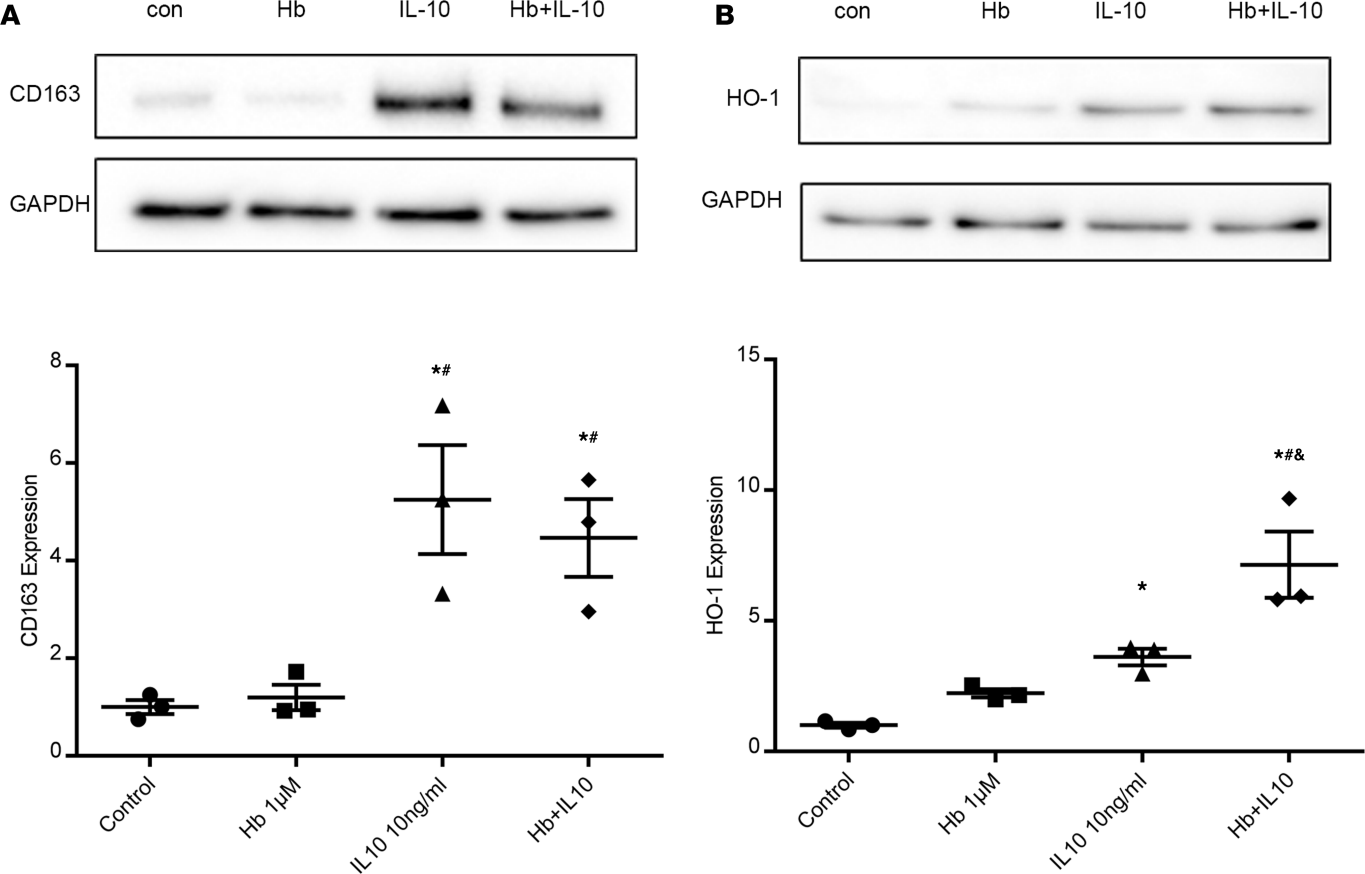
IL-10 induced CD163 and HO-1 expression in THP-1 macrophages cultured under hyperglycemia. THP-1 cells were differentiated to macrophages and cultured under high glucose conditions (25 mM). (**A** and **B**) Cells were treated as described in Methods, and CD163 (**A**) and HO-1 (**B**) protein expression were measured by Western blots. GAPDH was used as a loading control. Top: representative blots. Bottom: quantitative data. One-way ANOVA, followed by Tukey’s test; *n* = 3 per group, *P < 0.05 vs. control; ^#^*P* < 0.05 vs. Hb; ^&^*P* < 0.05 vs. IL10.

To further investigate the mechanism of IL-10 cardioprotection, we investigated the effect of IL-10 on macrophages in cell culture. The direct effect of IL-10 plus Hb on macrophages was tested under hyperglycemic conditions, and CD163 and HO-1 expression were measured. Differentiated THP-1 cells were cultured under hyperglycemic (25 mM glucose) conditions. As depicted in Figure 7A, IL-10 (10 ng/mL) or IL-10 plus Hb (1 μM) significantly increased CD163 expression in macrophages. Importantly, Hb alone did not induce CD163 expression. Next, induction of HO-1 was also observed when differentiated THP-1 cells were treated with IL-10 and a synergistic increase in HO-1 was seen with IL-10 plus Hb cotreatment under hyperglycemic conditions (Figure 7B). These findings were replicated in primary human CD14^+^ monocytes, and these experiments showed similar results (Supplemental Figure 3). Therefore, these data indicate that IL-10 can upregulate multiple components of the macrophage heme processing and clearance system.

### Effect of IL-10 on cardiomyocyte viability under ischemia/reoxygenation conditions.

**Figure 8:**
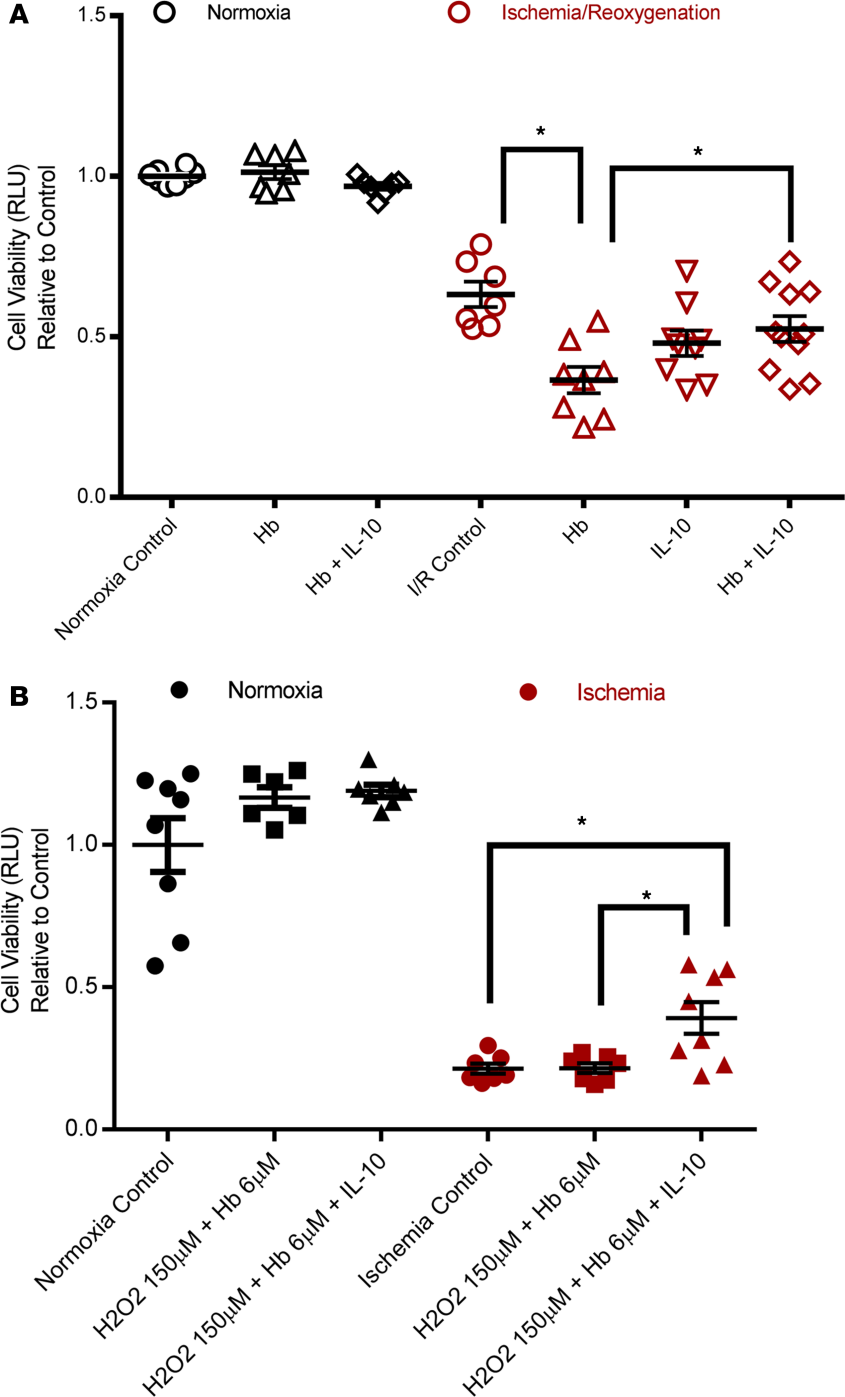
IL-10 protected against hemoglobin- and ischemia-induced injury in HL-1 cardiomyocytes. (**A**) HL-1 cells were treated with 6 μM Hb and/or 10 ng/mL IL-10 for 24 hours under normoxia or treated with 6 μM Hb and/or 10 ng/mL IL-10 under ischemia for 18 hours and reoxygenation for 6 hours. (**B**) HL-1 cells were treated with 6 μM Hb plus 150 μM H_2_O_2_ and/or 10 ng/mL IL-10 for 24 hours under normoxia or under ischemia for 24hours. Cell viability was measured by the end of the experiment. One-way ANOVA, followed by Tukey’s test; *n* = 8 per group, *P < 0.05.

We sought to determine if IL-10 has a direct protective effect on ischemic cardiomyocytes. The protective impact of IL-10 on diabetic ischemic cardiac injury could be a systemic or local phenomenon. Therefore, a direct effect of IL-10 on cardiomyocytes was assessed using the cardiomyocyte cell line HL-1. HL-1 cardiomyocytes were treated with Hb and/or IL-10 under normoxia and ischemia/reoxygenation (I/R) conditions. As depicted in Figure 8A, Hb caused cell death under I/R (*P* < 0.05 vs. I/R control) but not under normoxia (*P* > 0.05 vs. normoxia control). IL-10 protected cardiomyocytes from Hb-induced cell death under I/R (*P* < 0.05 vs. Hb).

Similar results were shown when cells underwent a “multi-hit” injury with ischemic conditions plus Hb and H_2_O_2_. As depicted in Figure 8B, IL-10 treatment significantly improved cell viability (*P* < 0.05 vs. H_2_O_2_ + Hb under ischemia), indicating direct cardioprotection of IL-10 against ischemic injury in cardiomyocytes.

## Discussion

The main findings of this study are that DM MI has greater plasma-free Hb and larger infarct size compared with nondiabetic MI. DM MI also has deposition of Hb and reactive iron within the infarct zone, as well as associated apoptosis and oxidative stress. IL-10 provides cardioprotection after MI in diabetic mice, and this effect is partially lost when mice were cotreated with SnPP. IL-10 treatment resulted in improved cardiac function and decreased infarct size. Furthermore, IL-10 treatment increased capillary density, reduced cardiomyocyte apoptosis, and reduced CD68^+^ cells in the infarct border zone after DM MI. Systemic IL-10 treatment increased HO-1 protein expression and decreased inflammatory gene expression in the infarcted heart. In addition, IL-10 induced multiple components of the heme clearance pathway.

These findings underscore the pathologic role of free heme after MI. Recent laboratory data indicate myocardial hemorrhage after MI, a previously underappreciated phenomenon (18, 19). Furthermore, clinical studies using cardiac MRI have demonstrated significant myocardial hemorrhage after MI (5, 6). In addition, in clinical studies, myocardial hemorrhage and persistent iron within the infract zone were associated with larger infarct size, worse left ventricular systolic function, and higher rates of death (7). We sought to determine whether IL-10 could provide cardioprotection in DM MI via upregulation of heme clearance mechanisms.

Heme proteins are ubiquitous and integral in many biological processes, especially oxygen handling and oxidative phosphorylation. Heme proteins contain the heme moiety, consisting of a porphyrin ring and an iron molecule, which is critical for the function of heme proteins in oxygen handling (20). Heme proteins are tightly regulated, and free heme has known cellular toxicity. After MI, cardiomyocyte cell death, microvascular destruction, and hemorrhage result in cell-free heme proteins entering the local microenvironment. Furthermore, cell-free heme proteins also enter the circulation after MI, as demonstrated in this study. These cell-free heme proteins function as DAMPs and activate inflammatory and oxidative pathways through a variety of mechanisms (9).

Prior studies have demonstrated that diabetic conditions are particularly maladapted to the presence of significant cell free heme due to downregulation of CD163, the Hb:haptoglobin scavenger receptor. Normally, the presence of cell free heme leads to upregulation of CD163 on macrophages and increased cellular processing of heme; however, diabetic conditions have impaired CD163 regulation (13).

IL-10 has significant effects on 2 key steps in the heme processing system. First, it upregulates CD163 in macrophages, increasing internalization of heme proteins into macrophages and clearance of free Hb (10, 11). Second, IL-10 also upregulates HO-1, the main intracellular enzyme responsible for degradation of heme proteins (12). Although HO-1 is known to be induced by ischemic injury, we have demonstrated that IL-10 results in much greater HO-1 expression within the infarcted myocardium compared with MI alone (Figure 3). HO-1 has been demonstrated to have protective effects in a number of pathological cardiac conditions, including MI (21). In summary, IL-10 may be particularly beneficial for MI in diabetic patients since it is able to upregulate multiple components of the heme clearance system and provides robust cardioprotection.

Although these data provide strong support for the protective effects of IL-10 after DM MI, along with support for the direct protective effect of IL-10 on ischemic cardiomyocytes, the use of pharmacologic treatment and chemical inhibitors is a limitation. The use of SnPP chemical inhibitor has well-known limitations, and there are concerns about a lack of specificity. Nonetheless, this study was undertaken as a preclinical study meant to replicate a clinical treatment paradigm. Overall, these findings support further exploration of IL-10 as a treatment during the acute phase of DM MI.

Taken together, our findings demonstrate that IL-10 provides cardioprotection in DM MI largely through upregulation of HO-1 and other aspects of the heme clearance system. These findings underscore the importance of free heme in mediating cardiac damage after DM MI and the ability of IL-10 to rescue the dysfunctional heme processing system in diabetes.

## Methods

See complete unedited blots in the supplemental material.

### Mice.

Male C57BL6 mice (stock no.000664) were obtained from The Jackson Laboratory. Mice were housed in a sterile barrier facility.

### Induction of diabetes.

Eight- to 10-week-old mice were given 5 daily i.p. injections of STZ (MilliporeSigma) dissolved in sterile citrate buffer (50 mmol/L sodium citrate, pH 4.5, 50 mg/kg b.w.) to induce diabetic conditions. Similarity, i.p. injection of citrate buffer was administered to control mice for 5 days. Fasting glucose levels were determined at baseline and then at 2 weeks after the last injection. Whole blood glucose levels were measured using a glucometer (CareTouch blood glucose monitoring system, Future Diagnostics LLC). Two weeks after the last STZ injection, fasting glucose levels were assessed. Mice with a fasting glucose level > 16.65 mmol/L (300 mg/dL) were considered diabetic and used for subsequent MI experiments.

### MI model.

Mice were subjected to MI by ligation of LAD coronary artery as described previously (17). Mice were anesthetized by 2%–3% isoflurane, orally intubated with a 22G i.v. catheter, and artificially ventilated with a respirator (Harvard Apparatus). To provide analgesia, buprenorphine SR (0.5 mg/kg) was injected s.c. before the operation. A left intercostal thoracotomy was performed, and the ribs were retracted with 5-0 polypropylene sutures to open the chest. After the pericardium was opened, the LAD branch of the left coronary artery was ligated distal to the bifurcation between the LAD and diagonal branch using 8-0 polypropylene sutures through a dissecting microscope. After positive end-expiratory pressure was applied to fully inflate the lung, the chest was closed with 7-0 polypropylene sutures and a 22G syringe was used to evacuate air from the chest cavity. The survival rate of MI surgery was 86%. The mice in the sham group underwent the same procedure except for the LAD ligation.

### Study design.

There were 2 sets of the study consisting of 36 and 45 mice, respectively. Sample sizes were determined by power analysis based on survival rate of MI and previous experience with this model. The first set of mice aimed to assess the outcomes of MI between diabetic mice and nondiabetic mice. Once diabetes was induced, mice were randomly assigned to 4 groups: control sham (*n* = 6), Con MI (*n* = 10), diabetic sham (*n* = 6), and DM MI (*n* = 9). Acute ischemic cardiac injuries were assessed 24 and 72 hours after MI. The second set of diabetic mice was used to assess the effect of IL-10 after MI. Diabetic mice were randomly assigned to 4 groups: sham (*n* = 5), vehicle control (MI + vehicle) (*n* = 11), IL-10 (MI + IL-10) (*n* = 11), and IL-10 plus Tin protoporphyrin (SnPP) (MI + IL-10 and SnPP) (*n* = 12). Surgeons were blinded to treatment assignment. IL-10 was obtained from R&D Systems, and SnPP was obtained from Frontier Scientific. Vehicle consisted of a sterile solution of 0.1% BSA in PBS. IL-10 was reconstituted in a sterile solution of 0.1% BSA in PBS. IL-10 was administered by s.c. injection at a dose of 50 μg/kg b.w. on days 0, 1, 3, 5, and 7 after MI (17). SnPP was administered by i.p. injection at a dose of 25 mg/kg on days 0, 1, 3, 5, and 7 after MI, as described previously (22).

### Echocardiography and invasive physiology.

Transthoracic echocardiography was performed with a VisualSonics (VEVO 770; VisualSonics Inc.). Systolic and diastolic LV dimensions and fractional shortening were measured at the midpapillary muscle level by M-mode tracings. Echocardiographic studies were conducted at day 0 (baseline) and at 7, 14, and 28 days after MI on mice anesthetized with a mixture of 1.5% isoflurane and oxygen (1 L/min).

At 28 days after infarction, animals were anesthetized with 1.5% isofluorane, and a micromanometer-tipped Millar catheter (Millar Instruments) was advanced into the left ventricle via the right carotid artery to obtain pressure-volume loops for measuring left ventricular end-diastolic pressure (LVEDP), left ventricular end-systolic volume (LVESV), +dP/dt.

### Measurement of infarct size.

TTC staining was used to assess myocardial tissue viability and determine myocardial infarct size in the acute setting. Mice were sacrificed at 24 hours after MI. The hearts were cut transversely 2 mm below the ligation suture and 1 mm thick. The tissue slices were incubated in 1% TTC PBS solution, pH 7.4, at 37°C for 20 minutes. Then, the slices were fixed in 10% PBS-buffered formalin for 15 minutes at room temperature. The infarcted area (white) and total LV area were measured using ImageJ software (NIH, version 1.52).

Masson’s trichrome staining was used to assess the infarct sizes in the chronic phase at day 28 after MI. The hearts were perfusion-fixed with 10% buffered formalin, embedded in paraffin, and then horizontally sectioned between the ligation and the apex. The myocardial infarct size was measured using the circumference method on Masson’s trichrome–stained tissue sections using ImageJ. The fibrotic infarct scar and total LV circumference were measured, and the ratio was expressed as the percentage of infarcted myocardium.

### Hb and heme scavenger protein measurements.

Okajima staining for Hb was carried out on 4-μm paraffin-embedded heart sections. Hb was stained by 7.7% Alizarin Red S., and the slices were counterstained in hematoxylin. NovaUltra Prussian blue stain kit (catalog IW-3010, IHC World) was conducted to detect ferric ion present in the 5 μm 10% formalin-fixed paraffin-embedded heart tissue sections, according to the instruction from the manufacturer. Plasma Hb was measured by Mouse Hb ELISA (E-90HM, Immunology Consultants Laboratory Inc.). Plasma hemopexin and haptoglobin were measured by mouse hemopexin ELISA (GWB-D5D320, Genway) and mouse haptoglobin ELISA (E-90HPT, Immunology Consultants Laboratory Inc.), respectively.

### Apoptosis analysis.

TUNEL staining was carried out on 4-μm thick paraffin-embedded sections by using cell death detection assay (Roche). TUNEL^+^ nuclei showed green color. Cardiac myocytes were identified using sarcomeric α-actinin antibodies (MilliporeSigma). DAPI staining was used to count the total number of nuclei and to colocalize TUNEL-stained nuclei. The index of apoptosis was calculated as the percentage of apoptotic myocyte nuclei to total number of nuclei.

Alternative measure of apoptosis was carried out to detect the activated caspase-3 in formalin-fixed paraffin-embedded heart sections by SignalStain apoptosis (cleaved caspase-3) IHC detection kit (catalog 12692, Cell Signaling Technology). Images were obtained using an Olympus whole slide scanning microscope with an original total magnification of 200×.

### Immunofluorescence microscopy.

Deparaffinized tissue sections were stained with anti–Hb α subunit antibody (catalog ab174536, Abcam) for assessment of Hb deposition in the infarct zone. Primary antibody staining was followed by Alexa Fluor 594 goat anti–rabbit IgG (catalog ab150080, Abcam) secondary antibody, and sections were examined with a fluorescent microscope (Nikon ECLIPSE TE200). Capillaries were visualized by injecting mice with BS-1 lectin (catalog L-1100, Vector Laboratories) 10 minutes before euthanasia, and then staining sections with anti-lectin primary antibodies (catalog AS-2104, Vector Laboratories) and Alexa 546–conjugated anti–goat IgG (catalog A-11056, Thermo Fisher Scientific) secondary antibodies. Capillary density was quantified by counting positively stained tubular structures at 10 randomly selected hpf per section.

Deparaffinized tissue sections were stained with anti-CD68 (clone FA-11, catalog MCA1957, Serotec; Bio-Rad) for inflammatory cell infiltration, followed by FITC goat anti–rat IgG (clone poly4054, catalog 405404, BioLegend) secondary antibody, and sections were examined with a fluorescent microscope (Nikon ECLIPSE TE200). Inflammatory cell infiltration was assessed at 10 randomly selected hpf in the border zone of infarcted myocardium and expressed as number per hpf.

Coimmunofluorescence staining was conducted in heart tissues by rat anti-CD68 (Fluo488) (catalog MCA1957, Bio-Rad) and rabbit anti-CD163 (catalog ab182422, Abcam) followed by donkey anti-rabbit (Alexa Fluor) (catalog ab150076, Abcam). The slices were mounted with Fluoroshield mounting medium with DAPI (catalog 104139, Abcam). Immunofluorescence was then visualized on a confocal microscope (TCS SP5 LCSM, Leica).

### Lipid ROS production measurement.

IHC staining of 4-HNE was conducted to measure the lipid peroxidation in formalin-fixed paraffin-embedded heart sections by cell and tissue staining kit (R&D systems Inc.). The samples were blocked with VisUBlock mouse on mouse blocking reagent (catalog VB001, R&D Systems) and then incubated with primary antibody, 4-NHE antibody 1:500 (catalog MAB3249, R&D Systems).

### Western blot analysis.

Tissue or cell lysates were prepared using ice-cold radio immunoprecipitation assay buffer (RIPA; 158 mM NaCl, 10 mM Tris-HCl, pH 7.2, 1 mM ethylene glycol tetra-acetic acid [EGTA], 1 mM sodium orthovanadate, 0.1% SDS, 1.0% Triton X-100, 1% sodium deoxycholate, 1 mM phenylmethylsulfonyl fluoride). Proteins (50 μg) were electrophoresed and analyzed using anti-CD163 (catalog ab182422, Abcam) and anti–HO-1 (catalog ADI-OSA-110-D, Enzo Life Sciences Inc.). GAPDH (catalog sc-20357, Santa Cruz Biotechnology Inc.) was used as the loading control for each sample.

### Quantitative PCR.

Gene expression levels of *Il1b*, *Il6*, and monocyte chemoattractant protein 1 (*MCP1*) were quantified in the border zone of infarct as described previously (17). Total RNA was extracted from left ventricle with RNA-STAT60 (Tel-Test Inc.). Total RNA was reverse transcribed with iScript cDNA Synthesis Kit (Bio-Rad), amplification was performed using TaqMan 7300 (Applied Biosystems). Relative mRNA expression of target genes was calculated with the comparative threshold cycle method and normalized to 18S rRNA expression.

### Cell culture.

HL-1 cardiac muscle cells were purchased from MilliporeSigma. THP-1 human monocyte cells were obtained from ATCC. THP-1 cells were differentiated into macrophages by 100 ng/mL PMA for 2 days in RPMI 1640 medium. Then, THP-1 cells were cultured in hyperglycemic (25 mM) DMEM with 10% FBS during the treatment.

Frozen human peripheral blood CD14^+^ monocytes were purchased from Physicians Plasma Alliance (PPA). Monocytes were differentiated into macrophages in the presence of 50 ng/mL M-CSF (216-MC-025, R&D Systems) for 6 days in DMEM medium (catalog 11995073, Thermo Fisher Scientific) supplemented with 10% FBS (catalog S11150, Atlanta Biologicals) and 1% penicillin-streptomycin (catalog 15-140-122, Fisher Scientific). Then, the monocyte-derived macrophages were cultured in hyperglycemic (25 mM) DMEM with 10% FBS and 1% penicillin-streptomycin during treatment.

### Cell viability assay.

Cell viability was measured using CellTiter-Glo Luminescent Cell Viability Assay kit (catalog g7570, Promega) detected by BMG FLUOstar Omega Microplate reader. HL-1 cells were seeded at the density of 5000 cells per well on a 96-well plate and cultured with HL-1 expansion media (MilliporeSigma) for 24 hours. The cells were treated with 6 μM of Hb and/or 10 ng/mL of IL-10 in normoxia condition for 24 hours or in I/R conditions, which consisted of αMEM (no glucose) in a hypoxia chamber (94% N2, 5% CO_2_, and 1% O_2_) for 18 hours and then placed in normoxia condition for 6 hours of reoxygenation.

### Statistics.

Normality was tested by Kolmogorov-Smirnov in IBM SPSS statistics version 24. If the data met the test of normality, the mean ± SEM was reported and the parametric analysis was conducted. For comparisons between 2 groups, a 2-tailed independent *t* test was conducted. A 1-way ANOVA was used for the comparisons among 3 or 4 groups and followed by the post hoc analysis when ANOVA had an overall *P* value of < 0.05. Comparisons for measurements taken at multiple time points were assessed by a 2-way repeated measures ANOVA in order to assess differences related to both treatments (mice receiving vehicle, IL-10, or IL-10 plus SnPP) and time periods (baseline, day 7, day 14, day 28) followed by the Holm-Šidák post hoc test. If the data did not meet the normality criteria, then the median was reported and nonparametric analysis was conducted. Statistical analysis was conducted using GraphPad Prism 7 with statistical significance set at *P* < 0.05.

### Study approval.

This study was carried out in strict accordance with the recommendations in the NIH *Guide for the Care and Use of Laboratory Animals* (2011). The protocols were approved by the IACUC at Northwestern University and the University of Toledo.

## Author contributions

RG contributed designing research studies, conducting experiments, acquiring data, analyzing data, and writing the manuscript. LL contributed designing research studies, conducting experiments, acquiring data, analyzing data, and writing the manuscript. XZ contributed conducting experiments, acquiring data, and analyzing data. XF contributed conducting experiments. PK contributed conducting experiments, acquiring data, analyzing data, and writing the manuscript. SV contributed conducting experiments, acquiring data, analyzing data, and writing the manuscript. J. Tongers contributed designing research studies, analyzing data, and writing the manuscript. SM contributed conducting experiments, acquiring data, and analyzing data. NA contributed conducting experiments, acquiring data, and analyzing data. HS contributed conducting experiments and acquiring data. J. Tian contributed conducting experiments and providing reagents. RK contributed designing research studies, conducting experiments, acquiring data, analyzing data, and writing the manuscript.

## Supplementary Material

Supplemental data

## Version 1 09/03/2020

Electronic publication
