# Supplementary material for: IL-10 provides cardioprotection in diabetic myocardial infarction via upregulation of Heme clearance pathways
Source: JCI Insight. 2020 Sep 3;5(17):e133050. doi: 10.1172/jci.insight.133050 (PMC7526458; doi:10.1172/jci.insight.133050)
Supplement: Supplemental data [file jciinsight-5-133050-s009.pdf]

Supplementary Figure 1

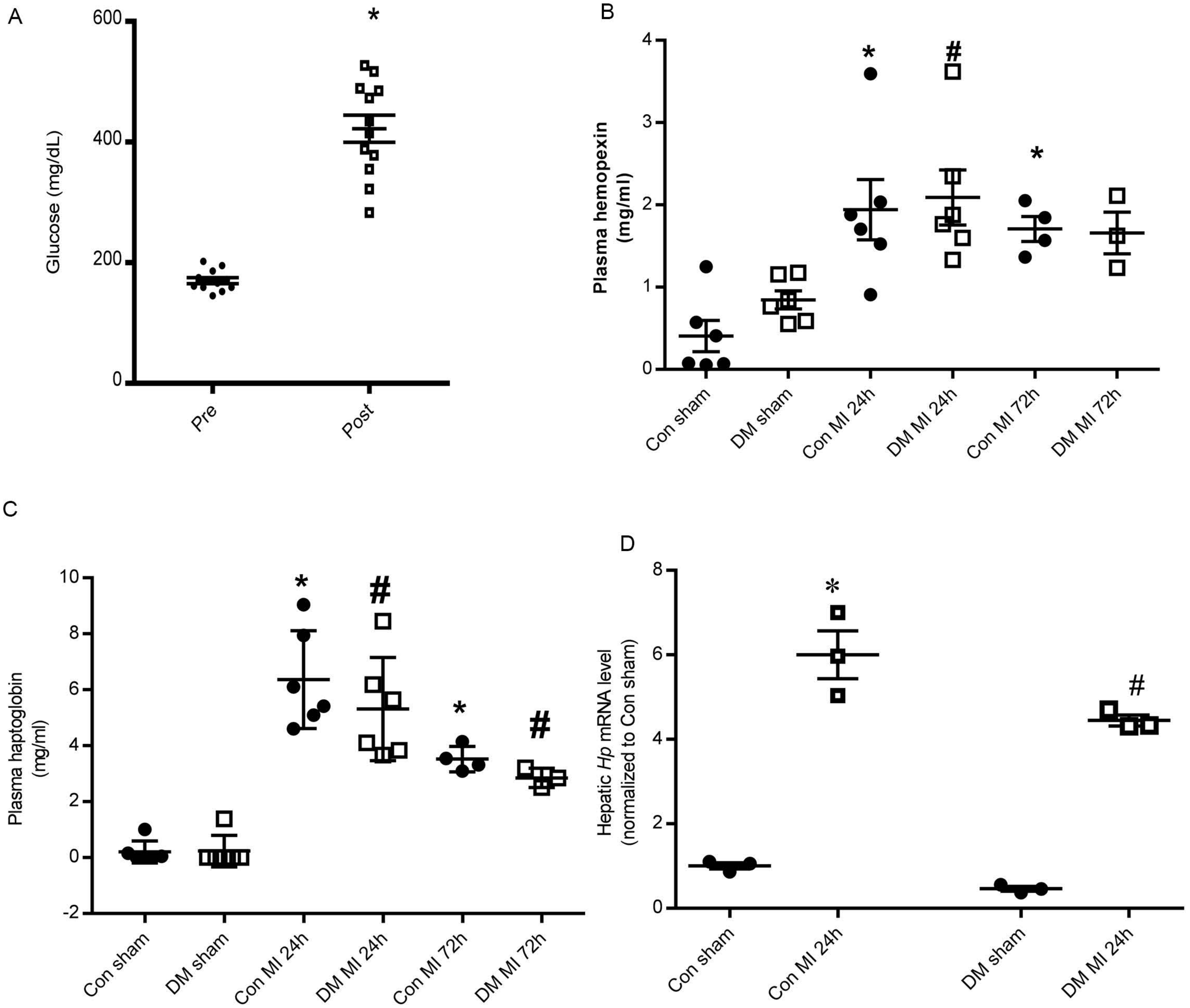

Supplementary Figure 1. Blood biochemistry in streptozotocin (STZ)-induced MI diabetes mouse model. A, C57/B6 mice were administrated 50 mg/kg STZ daily by intraperitoneal injections for 5 days. Fasting glucose levels were measured before (pre) and 2 weeks after the last STZ dose (Post). n=12 per group, t-test, *p<0.05 vs pre. B, Plasma hemopexin was measured by ELISA post-MI, C, Plasma haptoglobin was measured by ELISA post-MI. D, Hepatic Hp mRNA was measured by RT-PCR post-MI. One-way ANOVA, followed by Tukey's test. *p<0.05 vs Con sham; #p<0.05 vs DM sham.

Supplementary Figure 2

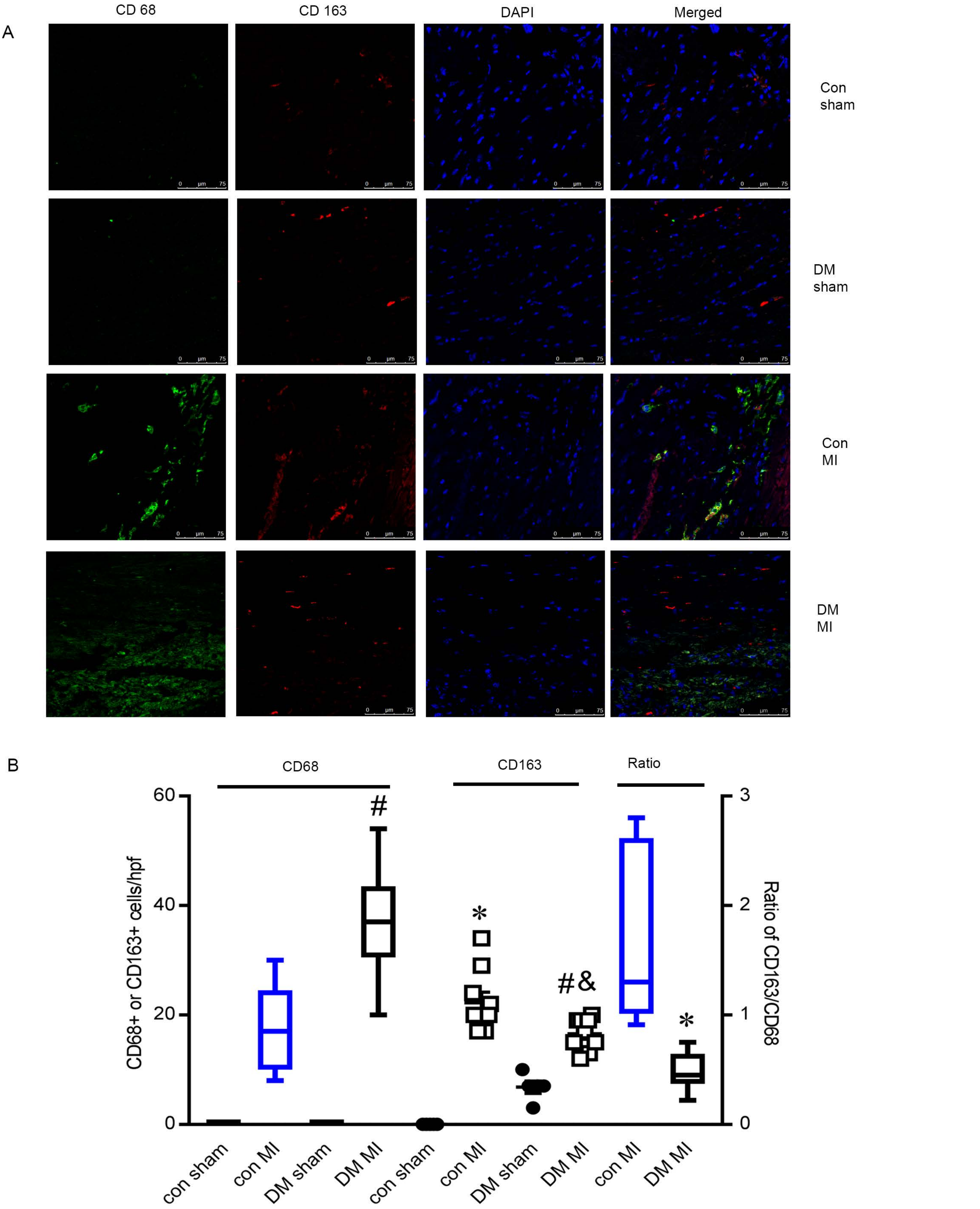

Supplementary Figure 2. Microscopic examination of CD68, CD163 in the border zone of the LV infarct on day 3 post-MI in diabetic mice. A, Confocal microscopic images of immunostaining of CD 68 (green), CD163 (red) and nuclei (DAPI, blue). CD 68 was greatly increased in post-MI hearts. B, Quantitative data of CD68 and CD163 positive cells in heart sections andCD163/CD68 ratio (degree of M2 polarization). Scale bar: 75μm. C, Quantitative data. Counts of CD68 and CD163 follow left Y axis. Ratio of CD163/CD68 follows right Y axis. One-way ANOVA, followed by Tukey's test was conducted to compare the numbers of CD68 in different group. Kruskal-Wallis test, followed by Dunn's test was used to compare the numbers of CD163 in different groups. Mann-Whitney test was conducted to compare the group difference of CD163/CD68 ratio between control mice and diabetic mice. n=5 (con sham and DM sham), n=9 (con MI and DM MI). *$p<0.05$ vs con sham; #$p<0.05$ vs DM sham; &$p<0.05$ vs DM sham.

Supplementary Figure 3

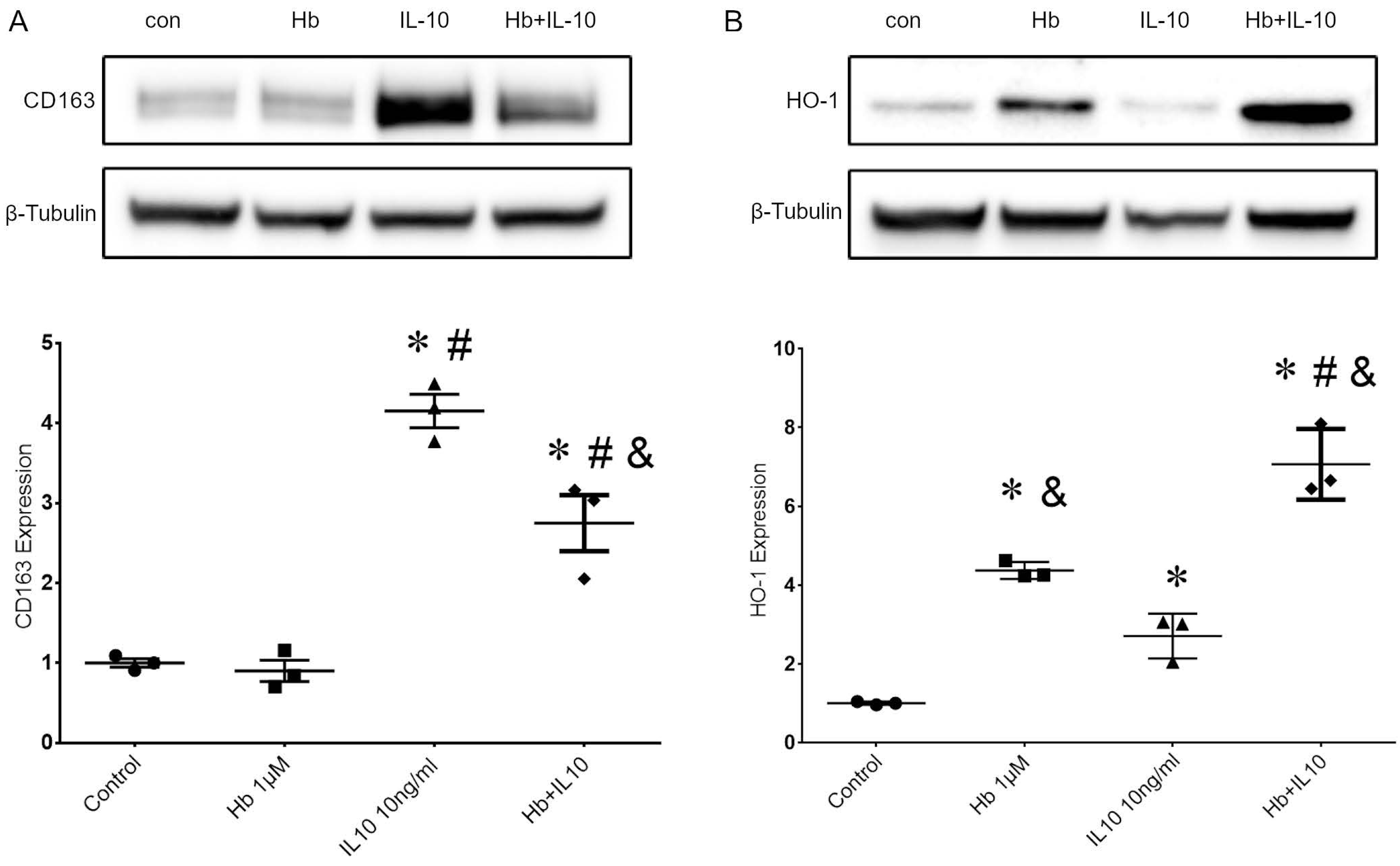

Supplementary Figure 3. IL-10 induced CD163 and HO-1 expression in primary human blood monocyte-derived macrophages cultured under hyperglycemia. Primary human blood monocytes were differentiated to macrophages and cultured under high glucose conditions (25mM). Cells were treated as described in the method and CD163 (A) and HO-1 (B) protein expression were measured by Western blots. β-Tubulin was used as a loading control. Top: Representative blots. Bottom: Quantitative data. One-way ANOVA, followed by Tukey's test, n=3 per group, *$p < 0.05$ vs Control; #$p < 0.05$ vs Hb; &$p < 0.05$ vs IL10.
